# Cytotoxicity and Pro-Apoptotic, Antioxidant and Anti-Inflammatory Activities of Geopropolis Produced by the Stingless Bee *Melipona fasciculata* Smith

**DOI:** 10.3390/biology9090292

**Published:** 2020-09-15

**Authors:** Josianne Rocha Barboza, Francisco Assis Nascimento Pereira, Renan Amphilophio Fernandes, Cleydlenne Costa Vasconcelos, Maria do Socorro de Sousa Cartágenes, Alberto Jorge Oliveira Lopes, Andreia Cristina de Melo, Isabella dos Santos Guimarães, Cláudia Quintino da Rocha, Maria Nilce de Sousa Ribeiro

**Affiliations:** 1 Laboratório de Farmacognosia, Departamento de Farmácia, Campus Bacanga, Universidade Federal do Maranhão, Av. dos Portugueses, 1966, São Luís 65080-805, Maranhão, Brazil; franciscopho2015@gmail.com (F.A.N.P.); lopesajo@gmail.com (A.J.O.L.); mnsousaribeiro@gmail.com (M.N.d.S.R.); 2 Programa de Pós-Graduação em Farmacologia e Química Medicinal, Instituto de Ciências Biológicas, Universidade Federal do Rio de Janeiro, Rio de Janeiro 21041-250, Rio de Janeiro, Brazil; renan_yako@hotmail.com; 3 Laboratório de Estudo Experimental da Dor, Campus Bacanga, Universidade Federal do Maranhão, Av. dos Portugueses, 1966, São Luís 65080-805, Maranhão, Brazil; cleydlenne@yahoo.com.br (C.C.V.); scartagenes@gmail.com (M.d.S.d.S.C.); 4 Divisão de Pesquisa Clínica e Desenvolvimento Tecnológico, Instituto Nacional de Câncer, Rua André Cavalcanti, 37, Rio de Janeiro 20231-050, Rio de Janeiro, Brazil; andreia.melo@inca.gov.br (A.C.d.M.); isasguimaraes@hotmail.com (I.d.S.G.); 5 Laboratório de Química de Produtos Naturais, Departamento de Química, Campus Bacanga, Universidade Federal do Maranhão, Av. dos Portugueses, 1966, São Luís 65080-805, Maranhão, Brazil

**Keywords:** natural products, antitumor activity, new anticancer agents, apoptosis pathway, molecular docking, drug discovery

## Abstract

Geopropolis is produced by some stingless bee species, such as *Melipona fasciculata* Smith, a native species from Brazil. This study aims to investigate the antioxidant and anti-inflammatory activities and cytotoxicity effects of geopropolis hydroethanolic extracts against lung (H460 and A549) and ovarian (A2780 and ES2) cancer cell lines and non-tumor (HUVEC) cell lines using chemical identification by LC/MS/MS analysis and in silico assays to determine which compounds are associated with bioactivity. The antioxidant activity of extracts and inhibitory activity against COX enzymes were assessed by in vitro assays; cytotoxicity effect was evaluated by the MTT assay; cell cycle was assessed by flow cytometry and apoptosis by Western blotting. The geopropolis extracts showed great radical scavenging potential, preferential inhibition of COX-2, decreased cancer cell viability, non-cytotoxic effects against the non-tumoral cell line, besides modulating the cell cycle and inducing cancer cell apoptosis through the activation of caspase-3 and PARP protein cleavage. The in silico study suggests that corilagin, typhaneoside, taraxerone and marsformosanone, identified by LC/MS/MS, can be associated with anti-inflammatory activity and cytotoxic effects. Thus, the current study suggests the potential of geopropolis concerning the research field of new pharmacological alternatives regarding cancer therapy.

## 1. Introduction

Cancer arises from the gradual accumulation of genetic alterations that increase cell proliferation [1]. It is the second main cause of death and has been recognized as one of the major public health problems worldwide. In 2018 alone, over 18 million new cancer cases were reported and over 9.5 million deaths by cancer were recorded worldwide, according to GLOBOCAN [2].

The gold standard treatment consists of the use of chemical neoplastic agents such as alkylating agents, antimetabolites, topoisomerase inhibitors and mitotic inhibitors which, in many cases, no longer present encouraging results and result in severe side effects [3]. Therefore, the development of new alternative drugs exhibiting low toxicity, high efficiency and the ability to prevent cell proliferation and/or promote apoptosis has become the major focus of cancer therapy in recent years [4,5].

In addition to apoptosis-inducing drugs, anti-inflammatory agents selective for cyclooxygenase 2 enzyme (COX-2) are traditionally reported be an effective adjuvant strategy for cancer therapy. COX-2 is involved in several malignant neoplasm processes, such as in the promotion of apoptotic resistance and in the proliferation, angiogenesis, inflammation, invasion and metastasis of cancer cells. Therefore, the use of COX-2 inhibitors is significant in managing metastasis risk reduction attempts in cancer patients while also resulting in higher susceptibility of cancer cells to gold standard treatments, such as radio and chemotherapy, resulting in better treatment efficiency [6].

Currently, the search for natural products exhibiting potential in cancer therapy has become prominent [7,8]. Geopropolis, a natural product derived from stingless bees, is noteworthy among natural products, displaying the highest potential in this regard.

Geopropolis is produced by stingless bees, formed by resinous material from plants collected by the bees, salivary bee secretions, wax, and clay or soil [9]. *Melipona* (*Melikerria*) *fasciculata* Smith 1858 (*Apidae*, *Meliponini*) is a stingless bee species cultivated for centuries by the indigenous population and small producers of Baixada (flooded fields) and Cerrado (Brazilian savannah) areas in Maranhão, a northeastern Brazilian state, to produce honey, geopropolis, wax and pollen [10,11].

Several geopropolis biological properties have been reported, including antinociceptive and anti-inflammatory [12,13,14], immunomodulatory [15,16], antimicrobial [14,17,18], antileishmanial [19] and antioxidant activities [19,20,21,22,23].

The antitumoral activity of *M. fasciculata* geopropolis has been previously evaluated against canine osteosarcoma (OSA) cells and cytotoxic effects have been reported in human leukemia monocytic cell lines [24,25]. However, the cytotoxic action of *M. fasciculata* geopropolis in other tumor cell lines has not yet been investigated.

In this context, this research aimed to evaluate the antioxidant, anti-inflammatory and cytotoxic activities of a hydroethanolic geopropolis extract of produced by *M. fasciculata*, identifying its chemical composition and correlating the identified compounds with detected biological activities through in silico assays and, finally, to contribute to the bioprospecting of new products exhibiting antitumor activity.

## 2. Materials and Methods

### 2.1. Geopropolis Samples

Two geopropolis samples of *M. fasciculata* Smith were collected in April 2018 being taken directly from the internal parts of a beehives located in meliponary in Viana (03°13′13″ S and 45°00′13″ W) and Pinheiro (02°31′17″ S and 45°04′57″ W) municipalities in the “Baixada” (flooded fields area, Brazil) from Maranhão State, northeast Brazil. After collection, the geopropolis samples were separated, identified, stored in a sterile recipient and kept at 4 °C until preparation of extract and further analysis. As determined by Brazilian legislation for research that uses the Brazil’s genetic heritage, this research is registered on National System of Genetic Heritage Management and Associated Traditional Knowledge (SISGEN) under code ABCEA59.

### 2.2. Extraction of Samples

The in natura geopropolis samples were processed as described by our research group in Dutra et al. [21]. The geopropolis was triturated until powder (200 g) and were individually extracted by maceration with 70% ethanol/water (70:30, *v*/*v*) for 6 days at a solid:solvent ratio of 1 to 5 (*w*/*v*), with solvent renewal after 72 h. The resulting product from extractions was combined, filtered, concentrated in a rotary evaporator under vacuum at 40 °C, and lyophilized, obtaining the hydroethanolic geopropolis extract (EHGV) (Viana sample) and EHGP (Pinheiro sample) and kept refrigerated until their use.

### 2.3. Determination of Antioxidant Activity

#### 2.3.1. DPPH• Radical Scavenging Activity

The antioxidant activity of hydroethanolic geopropolis extracts was evaluated by using the DPPH• free radical scavenging assay as described by Brand–Willians [26] with modifications as described by our research group in Dutra et al. [21]. The samples of geopropolis extracts were solubilized on methanol at concentrations (30–480 μg/mL) and added to a methanol solution of DPPH• (40.0 μg/mL). After 30 min of reaction at room temperature in the dark, the absorbance of each solution was read at 517 nm in a Lambda 35 UV–Vis spectrophotometer (PerkinElmer, Inc., Waltham, MA, USA). Methanol ACS was used as blank, and DPPH• solution was used as negative control. Trolox^®^ (positive control) standards were treated under the same conditions as the samples. The percent inhibition was calculated using the formula

DPPH• scavenging activity (%) = 100 − (A_sample_ − A_blank_) × 100/A_control_

where A_sample_ = absorbance of the sample after 30 min of reaction, A_blank_ = absorbance of the blank, and A_control_ = absorbance of the control.

The results were expressed as inhibitory concentration at 50% (IC_50_). All experiments were performed in triplicate.

#### 2.3.2. Ferric Reducing Antioxidant Power Assay (FRAP)

The method described by Benzie et al. [27] with some modifications as described by our research group in Dutra et al. [21] was used to assessment the antioxidant activity based on iron reduction using the FRAP assay, measuring the ferric-reducing ability of a sample in acid medium (pH 3.6) through the formation of an intense blue color as the ferric tripyridyltriazine (Fe^3+^–TPTZ) complex due reduction to the ferrous (Fe^2+^) form. The samples of hydroethanolic geopropolis extracts were solubilized in methanol at different concentrations (12.5–200 μg/mL). The absorbance of the reaction mixture was read at 593 nm in a Lambda 35 UV–Vis spectrophotometer (PerkinElmer, Inc., USA) using FRAP solution as a blank. The calibration curve was constructed using different concentrations of FeSO_4_·7H_2_O (0–2000 μM) (R^2^ = 0.9892), and the results are expressed as millimoles of Fe^2+^ per gram of sample. Trolox^®^ standard was set as positive control. The results were expressed as millimoles of Fe^2+^ per gram of sample. All experiments were done in triplicate.

#### 2.3.3. ABTS•^+^ Assay

The ABTS•^+^ method (2,2′-azinobis-3-ethylbenzotiazoline-6-sulfonic acid) was carried out as described by Re et al. [28] with modifications by our research group in Lopes et al. [11]. For formation of the ABTS radical, the 7 mM ABTS•^+^ solution was mixed with 2.45 mM of potassium persulfate solution. This mixture was maintained in a dark room for 16 h for the complete oxidation of ABTS and the generation of the highly stable chromophore cation radical 2,2′-azino-bis(3 ethylbenzothiazoline-6-sulfonic acid) (ABTS•^+^). The radical was diluted in 70% ethanol/water (70:30, *v*/*v*) to an absorbance of 0.700 ± 0.020 as read at 734 nm. Readings were performed by reacting 20–1000 μg/mL of hydroethanolic geopropolis extracts with the ABTS•^+^ solution. All studies were performed at least in triplicate, monitoring the decrease in absorbance for 6 min; reported results correspond to the % of remaining chromophores compared to conditions in the absence of antioxidants. The IC_50_ values were determined to each sample, using the formula

Scavenging ability (%) = (1 − A_sample_/A_blank_) × 100.

### 2.4. In Vitro COX Inhibition

The assay was performed according to the manufacturer’s recommendations (COX Colorimetric Inhibitor Screening Assay Kit—Item No. 701050—Cayman Chemical^®^, Ann Arbor, MI, USA), as previously described by our research group in Lopes et al. [11]. Using 96-well microplates, the geopropolis extracts were evaluated at three different concentrations (2, 10 and 50 μg/mL) against COX-1 and COX-2 isoforms. After reagents and plates preparations following the manufacturer’s kit data sheet, the colorimetric analysis was performed, using arachidonic acid as the substrate of the COX-catalyzed enzyme reaction, and the plates were read at 590 nm.

### 2.5. Cell Culture and Morphological Analysis

A panel of four cancer cell lines (H460, A549, ES2 and A2780) and non-cancer cell (HUVEC) were selected for this study. Non-small cell lung carcinoma (NSCLC) cell lines A549 and H460, and ovarian cancer cell lines ES2 and A2780 were maintained in RPMI-1640 (Gibco, New York, NY, USA) supplemented with 10% (*v*/*v*) fetal bovine serum (FBS) (Gibco, New York, NY, USA), 1% (*v*/*v*) stabilized with penicillin solution (100 units/mL) and streptomycin (100 μg/mL) (Gibco, New York, NY, USA) at 37 °C in a humidified atmosphere of 5% CO_2_. Human umbilical vein endothelial cells (HUVEC) were maintained in Dulbecco’s Modified Eagle Medium (DMEM) (Gibco, New York, NY, USA) supplemented with 10% (*v*/*v*) FBS (Gibco, New York, NY, USA), 1% (*v*/*v*) stabilized penicillin solution (100 units/mL) and streptomycin (100 μg/mL) (Gibco, New York, NY, USA) at 37 °C in a humidified atmosphere of 5% CO_2_. Short tandem repeat (STR) analysis was performed in all four cell lines to confirm cell line identity. The cell morphology was examined using an inverted Zeiss Observer Z1 microscope and images were captured using Axio-Vision Rel. 4.8 software (Carl Zeiss, Jena, Germany).

### 2.6. Cytotoxicity Activity

The 3-(4,5-dimethylthiazol-2-yl)-2,5-diphenyltetrazolium bromide (MTT) assays were used to measure the cytotoxic effects of (EHGV and EHGP) and cisplatin (CDDP) (LIBBS, SP, Brazil) on human cancer cell lines and non-tumoral cells. Stock solutions of 1000 mg/mL of EHGV and EHGP were dissolved in dimethyl sulfoxide (DMSO). Desired concentrations of each drug were prepared by dilution with culture medium before use. The cell lines were seeded (1 × 10^4^ cells/well) at 96-well plates and after 24 h, were treated with EHGV and EHGP for 48 h and 72 h. Cells were subsequently incubated with 20 μL of MTT solution (5 mg/mL) for 4 h. The plates were then centrifuged at 450× *g* and to allow solubilization of the formazan crystals. Then, 100 μL of DMSO was added to each well and spectrophotometric absorbance reading at a wavelength of 538 nm was performed using Flex-Station 3 (Molecular Devices Corporation, San Jose, CA, USA). As negative control for the experiments, we used the cells without treatment. We also treated cells with vehicle control (10% DMSO *v*/*v*). All experiments were performed in quadruplicates. The half maximal cell growth inhibitory concentrations (GI_50_) values were estimated using GraphPad Prism version 8 for Windows (GraphPad Software, La Jolla, CA, USA).

### 2.7. Cell Cycle and DNA Content Analysis by Flow Cytometry

The DNA content and cell cycle distribution of tumor cells treated with EHGV were determined by flow cytometry after propidium iodide staining. Cells were seeded onto 6-well plates, and after 24 h, were treated with EHGV. The cells were subsequently collected and washed in phosphate-buffered saline (PBS) followed by incubation with Nicoletti buffer (0.1% NP-40 (*w*/*v*), 0.1% sodium citrate (*w*/*v*), 200 μg/mL RNase and propidium iodide 50 μg/mL) at 4 °C for 30 min in the dark [29]. Doublets and debris were identified and excluded. The samples were acquired at the low flow rate and at least 20,000 cells were counted for each analysis. The distribution of cells in each phase of the cell cycle was displayed as histograms. The stained samples were analyzed with FACSCalibur flow cytometry (BD, San Jose, CA, USA) and the results were analyzed using FlowJo software (FlowJo, LLC, Glendale, CA, USA).

### 2.8. Western Blotting

To analyze apoptosis, cells were trypsinized and plated into a six-well plate, and after 24 h, treated with EHGV (15.62 and 31.25 μg/mL) and CDDP (10 μM) for 48 h. Then, cells were lysed in 2× radioimmunoprecipitation assay (RIPA) buffer supplemented with protease and phosphatase inhibitors. Protein concentration was determined using the DC Protein Assay kit (BioRad, Hercules, CA, USA). Equivalent amounts of protein (50 μg) were separated by SDS-PAGE. The gels were then transferred to polyvinylidene fluoride (PVDF) membranes and blocked with 5% skim milk. Membranes were then incubated with primary antibodies against cleaved poly (ADP-ribose) polymerase (PARP) (Cell Signaling Technology, Danvers, MA, USA), cleaved caspase-3 (Cell Signaling Technology, Danvers, MA, USA) and glyceraldehyde 3-phosphate dehydrogenase (GAPDH) (Cell Signaling Technology, Danvers, MA, USA). Before blocking, the membranes were incubated with 1% glutaraldehyde for 30 min for the analysis of cleaved caspase-3 expression [30]. Detection was visualized with the ECL prime reagent (GE Healthcare, Sao Paulo, Brazil) and the images were then captured on a ChemiDoc Imaging system (Biorad, Hercules, CA, USA) using Image Lab software (Biorad, Hercules, CA, USA).

### 2.9. LC–ESI/IT–MS/MS Analysis

The EHGV and EHGP were analyzed by LC–ESI/IT–MS/MS (LC–20AD Shimadzu, Kyoto, Japan) and a Phenomenex Luna C–18 (250 × 4.6 mm, 5 μm) column at 25 °C was used. The mobile phase used was Milli-Q water (Millipore) with 0.1% formic acid (eluent A) and methanol (eluent B). Elution was performed on a linear gradient of 0 min—10% B; 1–40 min—100% B; 40–60 min—100% B. EHGV and EHGP were diluted in methanol and 0.1% Milli-Q water of formic acid at the final concentration of 30 mg/mL and filtered through a nylon filter (0.22 μm, Allcrom Sao Paulo, Sao Paulo, Brazil). The samples volume injected into the system was 10 μL, with a flow rate of 1 mL/min and UV–Vis detection at 254 nm. The LC was coupled to a mass spectrometer (Amazon Speed ETD, Bruker, MA, USA) equipped with electrospray ionization (ESI) and an ion trap (IT) type analyzer in negative mode under the following conditions: 4.5 kV capillary voltage, capillary temperature of 325 °C entrainment gas (N_2_) flow 12 L/min, nitrogen nebulizer at a pressure of 27 psi. The acquisition range was *m*/*z* 100–1000, with two or more events. The compounds were identified on the basis of the molecular ion mass fragmentation.

### 2.10. In Silico Assay

#### 2.10.1. Predictive Models and Theoretical Calculations

The metabolites identified in EHGV were schematically designed in 3D models on GaussView 5.0.8 [31] and had their geometric, electronic and vibrational properties optimized with Gaussian 09 [32] using the density functional theory (DFT) method, combining the hybrid functional B3LYP and the basis set 6-31 ++ G (d, p).

#### 2.10.2. Molecular Docking (MD)

All MD protocol utilized Autodock Vina [33]. The structure of the human cyclo-oxygenase 2 (COX-2) (PDB ID 5F19) and nuclear factor kappa-B (NF-κB) NEMO/IKKβ association (PDB ID 3BRV) and ligands were prepared for MD with AutoDock Tools, version 1.5.7 [34]. Docking methodology described in literature were used [35] with some modifications [36,37]. Gasteiger partial charges were calculated after addition of all hydrogens both in ligands, COX-2 and NEMO/IKKβ association structures. Non-polar hydrogens from COX-2, NEMO/IKK IKKβ and EHGV metabolites were subsequently merged. The dimensions of cubic box in the *x*-, *y*- and *z*-axes were 30 × 30 × 30. Grid box was centered on oxygen atom from residues Arg120 from COX-2 and Glu89 of NEMO domain. In addition to visual inspection, the initial coordinates of interaction complexes were chosen based on the criterion of better docking conformation of the lowest energy score.

### 2.11. Statistical Analysis

Statistical analyzes between groups were performed by analysis of variance (ANOVA) followed by Tukey test. The results that presented probability of occurrence of null hypothesis lower than 5% (*p* < 0.05) were considered statistically significant. Statistical analysis was performed using GraphPad Prism 8 software.

## 3. Results

### 3.1. Antioxidant Activity

The results show that EHGV had the highest antioxidant activity with DPPH IC_50_ (76.16 ± 1.05 μg/mL). In the FRAP assay, EHGV also showed a higher ferric reduction (2.91 ± 0.12 mmol Fe^2+^/g) compared to EHGP (1.10 ± 0.25 mmol Fe^2+^/g). Regarding the ABTS•^+^ IC_50_, EHGV was also the sample with high antioxidant activity (13.28 ± 0.11 μg/mL) (Table 1). All differences found were statically significant (*p* < 0.05).

### 3.2. COX Inhibition Assay

**Figure 1:**
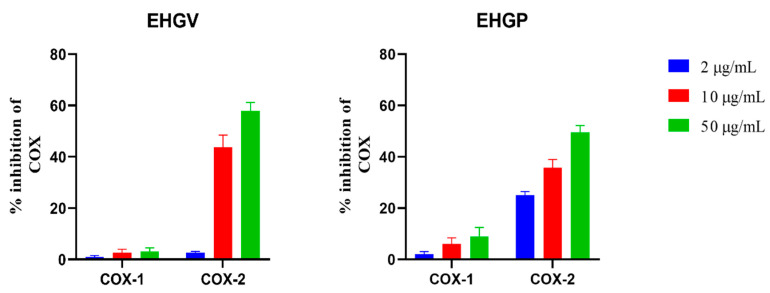
Percentual in vitro inhibition of COX-1 and 2 produced by hydroethanolic geopropolis extracts produced by *M. fasciculata* stingless bee was obtained in Viana (EHGV) and Pinheiro (EHGP) cities, Maranhão State, Northeast of Brazil.

Both EHGV and EHGP were tested for their ability to inhibit COX-1 and COX-2 enzymes. EHGV showed high COX-2 inhibitory activity (44% and 61% at 10 and 50 μg/mL, respectively) with less than 5% inhibition of COX-1 at 50 μg/mL. EHGP shown a minor potential for COX-2 inhibition at the same concentrations and had COX-1 inhibition next to 10% (Figure 1).

### 3.3. Cell Culture and Morphological Analysis

**Figure 2:**
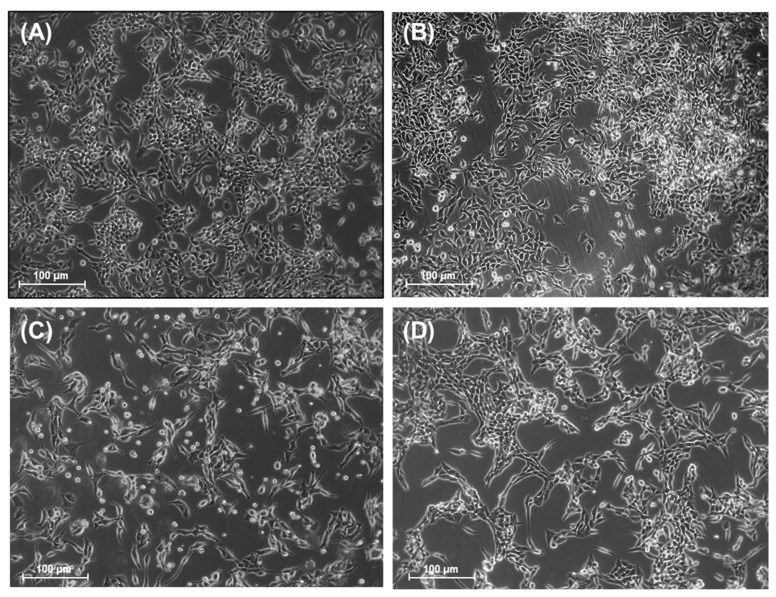

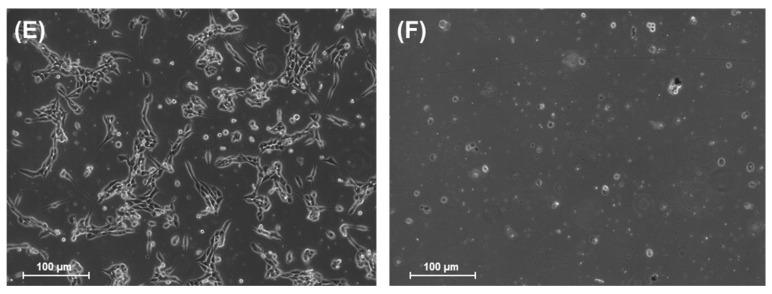
Analysis of morphological changes in A2780 tumor cells treated with EHGV. (**A**) Untreated A2780 control cells; (**B**) A2780 cells treated with vehicle (10% (*v*/*v*) DMSO); (**C**) A2780 cells treated with CDDP 10 μM; (**D**–**F**) A2780 cells treated with 15.62, 31.25 and 62.5 μg/mL EHGV, respectively. Cells were exposed to various concentrations of EHGV, CDDP and DMSO vehicle control and morphological changes were observed following 48 h of treatment. The cells were photographed (magnification 10×) with Axio-Vision Rel. 4.8 software. Scale bar = 100 μm.

To assess the antitumoral activity of EHGV, we firstly treated A2780 ovarian cancer cells with 15.62, 31.25 and 62.5 μg/mL EHGV for 48 h. Morphological differences were observed between the EHGV-treated, control cells and cells treated with 10% DMSO (*v*/*v*) (vehicle) (Figure 2A,B vs. Figure 2D–F). It is possible to observe that after treatment with EHGV, the cells become rounded and shrunken and detached themselves from the substrate. These morphological changes were absent in control cells and cells treated with vehicle (Figure 2). Morphological changes are valuable for determining the preliminary potential of anticancer activity of EHGV. As positive control, we used CDDP (10 μM) at a clinically relevant concentration [38] (Figure 2C).

### 3.4. Cytotoxic Activity

Then, we assessed the cytotoxic activity of EHGV and EHGP in lung cancer (A549 and H460), ovarian cancer (ES2 and A2780) and non-tumoral (HUVEC) cell lines treated with increasing concentrations of EHGV and EHGP for 48 and 72 h.

**Figure 3:**
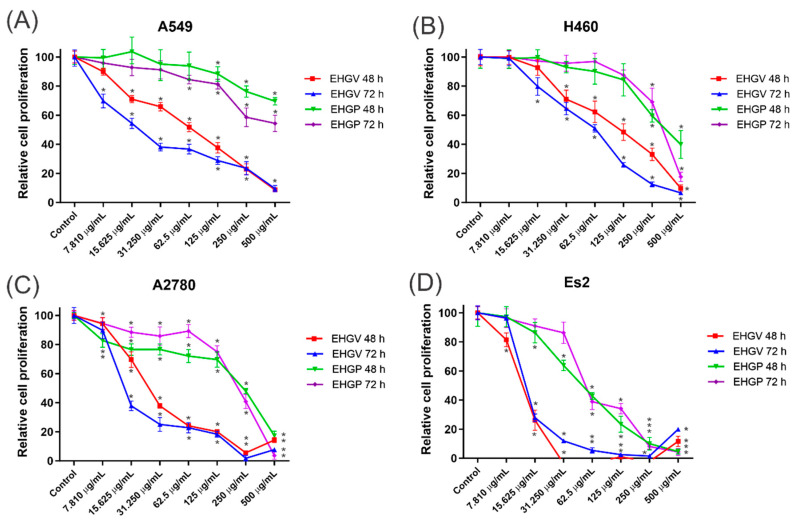

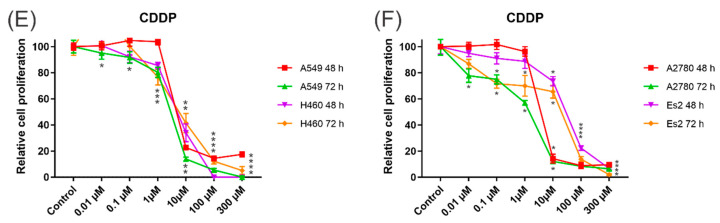
Effects of EHGV and EHGP in A549 (**A**), H460 (**B**), A2780 (**C**), Es2 (**D**) and CDDP (**E**,**F**) in four cancer cell lines at 48 and 72 h with statistical results. 2-way ANOVA with Tukey post-test. (* indicates *p* ≤ 0.05; vs. control).

The results obtained through the MTT assay revealed that the extracts decreased the percentage of cell viability for ovarian (A2780 and ES2) and lung (A549 and H460) cancer cells in a dose- and time-dependent manner. The EHGV and EHGP extracts showed greater cytotoxicity in the highest concentrations evaluated and in the longer incubation time with the extract. EHGV extract demonstrated high cytotoxic effect in the 48 and 72 h time compared to EHGP. Among the seven concentrations used, it was observed that from 31.25 μg/mL, the EHGV extract already demonstrated the capacity to inhibit percentage growth (Figure 3).

**Figure 4:**
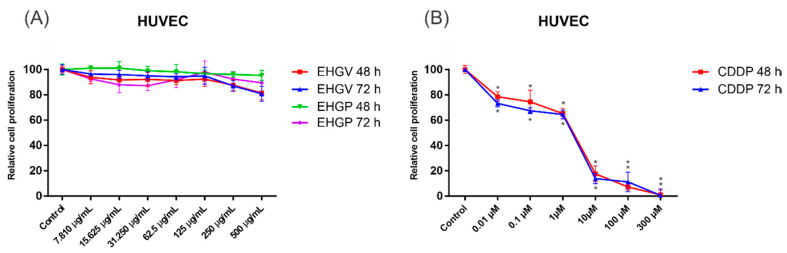
Effects of EHGV and EHGP (**A**) and CDDP (**B**) in non-tumor cells, HUVEC at 48 and 72 h with statistical results. 2-way ANOVA with Tukey post-test (* indicates *p* ≤ 0.05; vs. control).

Due to the high cytotoxicity for normal cells and numerous side effects caused by most of the traditional chemotherapy drugs used nowadays, we evaluated the cytotoxicity effects of EHGV and EHGP extracts against non-malignant HUVEC cells. As observed in Figure 4A, treatments with EHGV and EHGP for 48 and 72 h showed a non-significant influence on the cell viability of HUVEC cells. EHGV and EHGP at the most elevated concentration (500 μg/mL) for 72 h barely maintained cell viability in more than 80% (80.85 ± 5.90% and 89.54 ± 6.49%, respectively) of HUVEC cells. Importantly, when HUVEC cells were treated with CDDP, an extensively used chemotherapeutic drug for lung and ovarian cancer, we observed a decrease in cell viability in 48 and 72 h. We highlight the treatment with 10 μM, considered a clinically relevant concentration of CDDP, that markedly reduced cell viability close to 80% (only 17.61 ± 6.20% and 13.87 ± 4.01% of cell viability) in 48 and 72 h, respectively (Figure 4B). Collectively, these findings show that the present extracts exerted significant cytotoxic effects on cancer cells while showing low toxicity against non-malignant HUVEC cell lines.

The values for cell growth inhibition (GI_50_) of the EHGP and EHGV extracts were determined individually by the MTT assay over 48 and 72 h in the A2780, Es2, A549, H460 and HUVEC cell lines and are shown in Table 2. The results show that the EHGV extract significantly inhibited A2780 and A549 and was the most potent extract with an GI_50_ value of 16.92 μg/mL for A2780 and 22.64 μg/mL for A549 (Table 2).

### 3.5. Cell Cycle Analysis by Flow Cytometry and Analysis of Apoptosis by Western Blot

**Figure 5:**
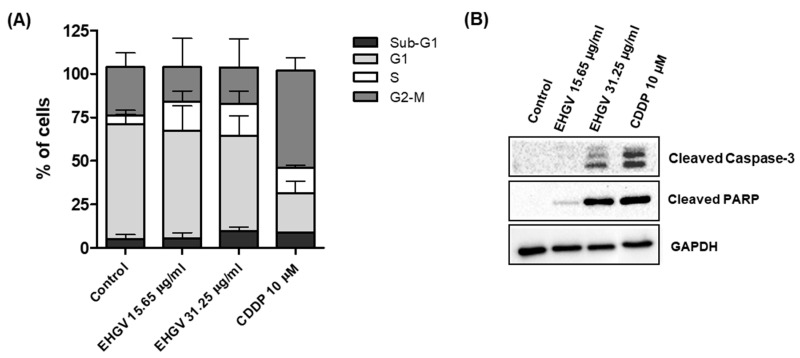
Analysis of effect of EHGV on cell cycle phase distribution and Western blot analysis of apoptosis-related proteins in A2780 cells treated with EHGV (15.65 and 31.25 μg/mL) and CDDP (10 μM) for 48 h. (**A**) Distribution of cells in sub-G1, G1, S or G2/M phases of cell cycle in A2780 cells treated with EHGV (15.65 and 31.25 μg/mL), CDDP (10 μM) and vehicle (control) for 48 h. (**B**) Western blot analysis of cleaved caspase-3 and cleaved PARP in A2780 cells treated with EHGV (15.65 and 31.25 μg/mL), CDDP (10 μM) and vehicle (control) for 48 h. GAPDH was used as loading control.

To explore the possible mechanisms underlying EHGV cytotoxicity in cancer cells, we analyzed cell cycle distribution and induction of apoptosis thought of cleaved caspase-3 and cleaved PARP in A2780 cells. Cell cycle distribution was analyzed by flow cytometry after a 48 h exposure to EHGV (15.65 and 31.25 μg/mL). EHGV treatment increased cells in S-phase compared with control cells treated with vehicle (Figure 5A). Additionally, treatment with EHGV at 31.25 μg/mL led to the accumulation of small DNA fragments in the sub-G1 phase (hypodiploid peak) in A2780 cells compared to control cells (5% vs. 9.79%). Interestingly, the treatment with CDDP, a drug currently employed in the treatment of ovarian cancer, showed the equivalent results in A2780 cells (8%) (Figure 5A).

Intrigued with the low proportion of cells in the sub-G1 phase (considered as apoptotic cells) and the impressive results in decreasing the percentage of cell viability in ovarian cancer cells (Figure 3), we next verified apoptosis-related proteins by Western blot. Cleavage of caspase-3 (17 kDa subunit) and PARP (89 kDa subunit) to their active forms is an important event in cancer cell apoptosis. As shown in Figure 5B, treatment with EHGV for 48 h increased the expression of cleaved caspase-3 and cleaved PARP in a dose-dependent manner. Additionally, A2780 cells treated with CDDP 10 μM for 48 h also showed an increase in these apoptosis-related proteins.

### 3.6. LC–ESI/IT–MS/MS Analysis

Table 3 and Table 4 summarize the list of identified compounds in the classes of glycosylated flavonoids, triterpenes, triterpenoid saponins, hydrolyzable tannins, anthraquinones and catechins, their retention time, molecular weight, molecular ions [M − H]^−^ and main ions of the products obtained by LC–ESI/IT–MS/MS for the 23 peaks of fragmentation of EHGV and EHGP.

### 3.7. In Silico Study

In the molecular docking study, all metabolites identified in EHGV were used. All compounds showed very satisfactory affinity parameters to both COX-2 and NF-κB structures. On COX-2, high parameter values were found for corilagin, typhaneoside and β-amyrin, with values for free binding energies of −9.3, −8.8 and −8.7 kcal/mol, respectively. Regarding NF-κB, taraxerone, marsformosanone and β-amyrin had parameters indicating high interaction, with free binding energies of −8.4, −7.7, −7.4 kcal/mol, respectively. The results of the free binding energy parameters of EHGV metabolites are shown in Table 5.

**Figure 6:**
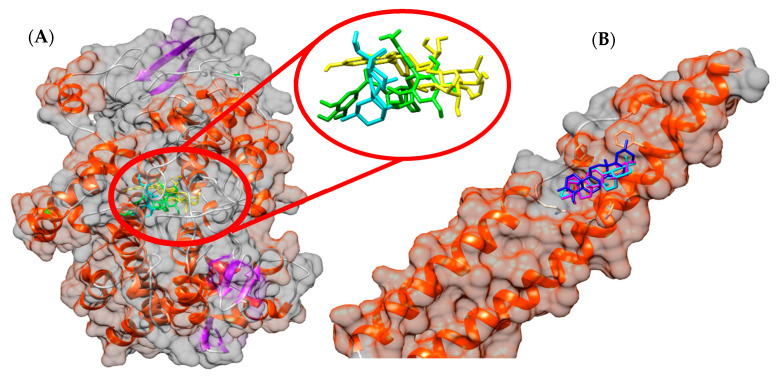
Spatial conformations obtained by molecular docking of corilagin (in green), typhaneoside (in yellow) and β-amyrin (in cyan) on COX-2 active site (**A**) and conformations of taraxerone (in blue), marsformosanone (in magenta) and β-amyrin (in cyan) on NEMO/IKKβ structure (**B**).

The docking results showed that corilagin, typhaneoside and β-amyrin have hydrogen bonds and van der Waals interactions with residues from COX’s catalytical site triad (Arg120, Thr385 and Glu524) and neighboring residues. It is also shown that the taraxerone, marsformosanone and β-amyrin performs van der Waals interactions with residues Glu89, Lys90, Leu93, Met94 and Phe97 (NEMO domain) and Glu729, Gln730, Ser733 and Phe734 (IKKβ domain). The spatial conformation from ligands obtained by the molecular docking study is shown on Figure 6.

## 4. Discussion

The findings reported herein indicate that EHGV and EHGP exhibit the ability to reduce ferric ions to ferrous ions (FRAP) and free radical scavenging activity (DPPH• and ABTS•^+^), suggesting significant antioxidant activity (Table 1). These results are in agreement with previously reported findings from stingless bee geopropolis [21,39,40].

Several types of oxidative damage at the cellular level are avoided in biomolecules such as lipoproteins and/or DNA, due to the presence of antioxidants. In the absence of these compounds, the oxidative stress produced by an increased production of reactive oxygen species (ROS) is responsible for triggering a complex and wide cascade of biochemical events harmful to organisms and associated to several pathological and disease processes, including cancer [41,42]. In addition, the interactions of antioxidant compounds with ROS through the elimination of free radicals are implicated in reducing conditions triggered by oxidative stress, such as cancer and other inflammatory processes [43].

Considering the antioxidant activity of *M. fasciculata* geopropolis extracts, COX inhibition potential and antitumor activity were evaluated by an in vitro cytotoxicity assay, where anti-inflammatory and antioxidant activities can be associated to antitumor activity [43,44]. Due to its higher antioxidant activity, EHGV also exhibited major inhibitory activity, inhibiting 61% of COX-2 and only 5% of COX-1 at 50 μg/mL (Figure 1). EHGV also demonstrated higher cytotoxic effect, decreasing cell viability in ovarian (A2780 and ES2) and lung (A549 and H460) cancer cells (Figure 3). Extracts exhibiting antioxidant properties commonly act as enzyme inhibitors, i.e., COX, xanthine oxidase, lipoxygenase, phospholipase A_2_ and others. The prostaglandin metabolism mediated by COX plays a fundamental role in inflammatory processes and is important in carcinogenesis, tumor differentiation, tumor growth and in suppressing tumor immunity, contributing to cancer immunotherapy resistance to several types of tumors [6,45,46]. In addition, a possible carcinogenesis inhibition mechanism has been reported for COX-2 inhibition since this enzyme plays an important role in the activation of local growth factors that lead to neovascularization, inflammation and carcinogenesis [47]. Thus, products that inhibit COX-2, such as *M. fasciculata* geopropolis, exhibit the potential to integrate future preventive and therapeutic anticancer strategies.

Considering the importance of lung and ovarian cancers, both resulting in high mortality rates, usually detected in advanced stages and exhibiting significant chemoresistance [2,48], two lung cancer cell lines (A549 and H460) and two ovarian cancer cell lines (ES2 and A2780) with different genetic backgrounds were selected for antitumoral activity evaluation of *M. fasciculata* hydroethanolic geopropolis extracts.

Several morphological changes in A2780 ovarian cancer cells treated with EHGV were observed, especially at the highest exposed concentration (62.5 μg/mL) (Figure 2). EHGV-treated cells became rounded and shrunken, exhibiting decreased density and detaching themselves from the substrate. These features are suggestive of EHGV-induced cell death mediated through apoptosis. Changes in cell morphology were less prominent when treated with clinically relevant concentrations of CDDP, routinely used in lung and ovarian cancer treatment [48].

Furthermore, the MTT assay was used to measure the cytotoxic effects of the extracts in lung and ovarian cancer cell lines. Our findings indicate that EHGV demonstrated higher cytotoxic effects at 48 and 72 h compared to EHGP in all cancer cell lines (Figure 3).

Drug discovery of new cytotoxic agents that explores differences between cancerous and normal cells continues to be a public health demand. Therefore, anticancer agents are expected to exhibit minimum effects on non-tumor cells. For this reason, we also evaluated extract cytotoxicity in non-malignant HUVEC cell lines. No significant toxic effects were observed, reinforcing the safety of the geopropolis extract and emphasizing its ability to inhibit cell proliferation, promoting its antitumor activity (Figure 4). Our investigations regarding the toxic effects of the extract corroborate Barboza et al. [49], who evaluated acute EHGV and EHGP toxicity in a zebrafish toxicity model and reported very low toxicity.

Previous studies have reported that *M. fasciculata* geopropolis exerts cytotoxic effects in a human leukemia monocytic cell line with significative decreases in cell viability at high concentrations (50 and 100 μg/mL) [25]. Additionally, Cinegaglia et al. [24] reported that *M. fasciculata* geopropolis also exhibits cytotoxic activity against canine osteosarcoma (OSA) cells. In another study, geopropolis produced by *Melipona mondury* Smith exhibited significant antiproliferative activities against various tumor cell lines (mouse melanoma, B16-F10, human hepatocellular carcinoma, HepG2, human promyelocytic leukemia, HL-60 and human chronic myelocytic leukemia K562) and no cytotoxic effects against non-tumor cells [14].

According to the USA’s National Cancer Institute (NCI), crude extracts with GI_50_ < 30 μg/mL in the preliminary assay are considered promising cytotoxic agents against neoplastic cells [50,51]. Therefore, EHGV is a promising product due to a GI_50_ value of 16.92 μg/mL in A2780 cancer cell lines and 22.64 μg/mL in A549 cancer cell lines (Table 2). Regarding the four-human cancer-derived cell lines, the A2780 ovarian cancer cell line was selected to screen EHGV’s ability to induce apoptosis (programmed cell death) and its impact on the cell cycle due to a higher GI_50_ value of 16.92 μg/mL for A2780. The A2780 cell line was treated with EHGV at two concentrations (15.65 and 31.25 μg/mL) for 48 h and then analyzed by flow cytometry. EHGV-treated A2780 cells displayed an increased percentage of cells in the S-phase compared with control cells treated with the vehicle. As mentioned previously, CDDP is routinely employed in the treatment of ovarian cancer and exhibited similar effects concerning an increased number of cells in the sub-G1 phase compared to the EHGV (31.25 μg/mL) treatment in A2780 cells (Figure 5A). It is important to emphasize that no studies have characterized cell cycle modulation after geopropolis extract exposure in cancer cells.

Apoptosis, a form of programmed cell death, involves the activation, expression, and regulation of various proteins [52]. Clinically, the main goal in cancer therapy is cancer cell death. Caspase-3 is a central apoptosis effector, catalyzing the specific cleavage of many key cellular proteins, like nuclear protein poly (ADP-ribose) polymerase (PARP) [53,54]. The results of the present study also indicate that EHGV dose-dependently enhances A2780 cell apoptosis after 48 h of treatment by increasing the expression of cleaved caspase-3 and cleaved PARP (Figure 5B).

PARP plays an essential role in several cellular process, such as maintenance of genomic stability, DNA repair and apoptosis [55], and is a target of caspase-3 protease activity. Caspase-3 cleaves PARP into two fragments (89 and 24 kDa) during apoptosis. This cleavage is considered a useful hallmark of cell apoptosis [56]. As shown in Figure 5B, EHGV activates caspase-3 cleavage and, consequently, reduces proteins levels throughout the total PARP length in EHGV-treated cells. This can be accompanied by increased PARP expression, an apoptosis characteristic [57].

Following the positive results of decreased cell viability in lung and ovarian cancer cells, chemical composition characterization was performed. The LC–ESI/IT–MS/MS analysis indicates a chemical composition similarity between the two extracts related to glycosylated flavonoids and triterpenes (taraxerone). Triterpenes ursane (marsformosanone), oleanane (β-amyrin) and dammaren (dipterocarpol) skeleton and glycosylated triterpene saponin (3-[Xyl]-28-Glc-phytolaccagenin) were identified in EHGV, which were not detected in EHGP (Table 3 and Table 4). These findings corroborate studies concerning the chemical composition of *M. fasciculata* geopropolis from different areas, due to the predominance of substances of the polyphenolic classes (hydrolysable tannins and flavonoids) and triterpenes [19,21,40,58].

Yam-Puc et al. [59] identified thirteen pentacyclic triterpenes in a chloroform–methanol–propolis extract from *Melipona beecheii* including marsformosanone, taraxerone and β-amyrin, which were also identified in EHGV. Besides taraxerone, two other pentacyclic triterpenoid (marsformosanone and β-amyrin) and one dammaren triterpene (dipterocarpol) and one glycosylated triterpene saponin (3-[Xyl]-28-Glc-phytolaccagenin) were also identified in EHGV. The triterpenes marsformosanone and dipterocarpol and 3-[Xyl]-28-Glc-phytolaccagenin, a triterpene saponin, were identified for the first time in *Melipona fasciculata* geopropolis. Triterpenoids, in general, are commonly attributed to inhibition of NF-κB activation and signal transduction, cell proliferation, apoptosis, angiogenesis and mitochondrial dysfunction [8].

According to the EHGV anti-inflammatory and antitumor activity results, which suggest that COX-2 receptor and NF-κB play a role in these effects, molecular docking of the compounds identified in the EHGV against these targets was also performed (Figure 6).

The molecular docking results suggest that corilagin, a hydrolysable tannin, typhaneoside, glycosylated flavonol, and the triterpene β-amyrin exhibit the best affinity parameters to COX-2 structure (Table 5). Corilagin exhibited greater interaction and its antitumor and anti-inflammatory potential is described in the literature, which may act in suppressing COX-2 expression at the gene and protein levels, demonstrating that this molecule may inhibit the inflammatory process [60,61,62]. No records concerning typhaneoside COX inhibitor potential are available, but is recognized that this molecule can regulate IL-6 and TNF-α [63] and promote cell proliferation and decrease NO levels in HUVEC cells [64], suggesting anti-inflammatory activity and no cytotoxic effects. β-Amyrin also displays antitumor effects against HepG2 liver carcinoma cells, causing apoptosis, cell cycle disruption and activation of the JNK and p38 signaling pathways [65]. β-Amyrin reduces the gene expression of TNF-α, IL-1β, IL-6, PGE2, COX-2 [66] and exhibits high inhibition of PGE2 and IL-6 secretion and NF-κB activation in a concentration-dependent manner, being a promising molecule for the treatment of various inflammatory disorders [67].

COX-2 is the main enzyme acting in prostaglandin (PGH2) production through the conversion of arachidonic acid. This prostaglandin can later be converted into different prostaglandins such as PGE_2_, PGD_2_, PGF_2α_, and also into thromboxane A_2_. These inflammatory prostanoids are closely associated to rapid and disordered tumor growth, characteristic of malignant neoplasms, since they promote cell division, metastasis and angiogenesis, in addition to inhibiting cell apoptosis [68,69]. Thus, the fact that EHGV displays a preference for COX-2 inhibition is directly correlated to the antitumor capacity reported herein.

Regarding the NF-κB receptor, taraxerone, marsformosanone and β-amyrin exhibited the best affinity parameters in the molecular docking study, with their conformations as the best free binding energy in the same region (Table 5), interacting with important catalytic residues such as the Glu89 NEMO and Ser733 IKKβ domains [70]. Taraxerone, identified in both EHGP and EHGV, has been described in the literature as displaying antitumor potential, inhibiting cancer cell proliferation and colony formation in A549 lung cancer cells, revealing potent cytotoxic effects in a dose-dependent manner and characteristic of apoptosis [71]. Taraxerone has also been reported as an antioxidant and iNOS inhibitor [72]. No records regarding marsformosanone’s potential as an NF-κB inhibitor agent are available, so this is the first study to suggest this activity. Pentacyclic triterpenes have been described as potential NF-κB signaling pathway inhibitors [73,74,75]. Laszczyk [76] described that triterpenes with a lupano, oleanan or ursano skeleton, including β-amyrin, display antitumor activity against different modes of action. β-Amyrin shows anti-inflammatory and anti-apoptotic effects on hepatic fibrosis in male rats [77]. Additionally, Ghante and Jamkhande [8] reported that triterpenoids display the ability to inhibit NF-κB activation.

NF-κB regulates the transcription of anti-apoptotic genes, contributing to cancer cell escape from apoptosis [78]. NF-κB inhibition in experimental studies has shown promising results in enhancing apoptosis and potentiating antitumor agent effects [79]. In addition, NF-κB is pivotal in inflammatory responses. Therefore, inhibition of the NF-κB signaling pathway exhibits potential therapeutic application in cancer and inflammatory diseases [80,81].

**Figure 7:**
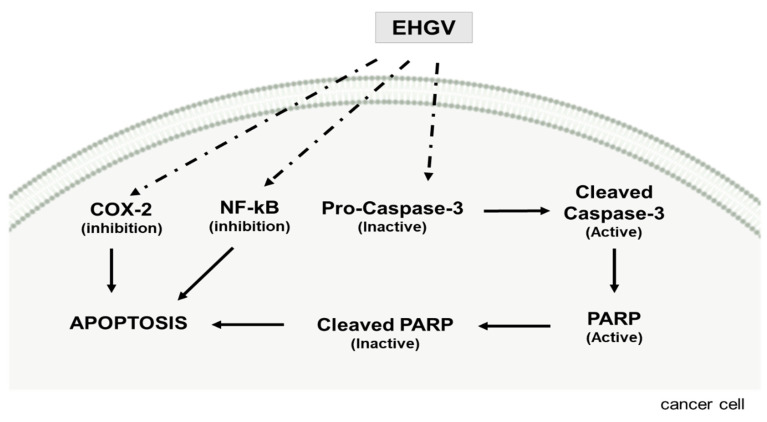
Proposed EHGV mechanism of action in ovarian cancer cells.

Thus, based on our EHGV result, an EHGV mechanism of action in ovarian cancer cells is suggested (Figure 7).

These findings reinforce our hypothesis that these molecules have the potential to become research targets for new drugs exhibiting anti-inflammatory and antitumor activities. Therefore, geopropolis may be a less toxic therapeutic alternative to be tested in the future in combination with monotherapy or polytherapy cancer treatment regimens.

## 5. Conclusions

The hydroethanolic geopropolis extract produced by *Melipona fasciculata* is composed of hydrolysable tannin, glycosylated flavonoids, anthraquinone, catechin, and triterpene substances which can be related to the antioxidant and anti-inflammatory activities and cytotoxic effects against A2780, ES2, A549, H460 cell lines. The extracts also have high preference for COX-2 inhibition, contributing effectively to antitumor activity. The in silico results, in concordance with our results from anti-inflammatory and antitumor activities, suggests that this activity can be due to COX-2 inhibition and NF-κB activation. Thus, we demonstrated for the first time that geopropolis produced by *M. fasciculata* has cytotoxic effects thought mediating apoptosis and cleaved caspase-3 activation in cancer cells, showing low toxicity against non-malignant HUVEC cell lines. We conclude that geopropolis is a natural product that exhibits anticancer properties that should be further evaluated in monotherapy or polytherapy schemes to improve chemotherapy–antitumor responses and long-term benefits in cancer patients.

## Figures and Tables

**Table 1:** Antioxidant activity (DPPH•, FRAP, ABTS^•+^) of hydroethanolic geopropolis extract produced by *Melipona fasciculata* Smith.

Sample	DPPH• IC_50_ (μg/mL)	FRAP (mmol Fe^2+^/g)	ABTS^+^ IC_50_ (μg/mL)
EHGV	76.16 ± 1.05 ^a^	2.91 ± 0.12 ^a^	13.28 ± 0.11 ^a^
EHGP	265.91 ± 0.29 ^b^	1.10 ± 0.25 ^b^	58.94 ± 0.09 ^b^
Trolox	3.01 ± 0.47	8.41 ± 0.28	3.69 ± 0.63

Values represent the mean of triplicate measurements ± standard deviation. Different letters in the same column indicate a significant difference (Tukey *p* < 0.05). EHGV—hydroethanolic geopropolis extract of *M. fasciculata* from Viana city, Maranhão State, Brazil; EHGP—hydroethanolic geopropolis extract of *M. fasciculata* from Pinheiro city, Maranhão State, Brazil; DPPH•: 2.2-diphenyl-1-picrylhydrazyl radical; FRAP: ferric reducing antioxidant power; ABTS•+: 2.2′-azinobis-3-ethylbenzotiazoline-6-sulfonic acid.

**Table 2:** Cell growth inhibition (GI_50_) in μg/mL of EHGP and EHGV for inhibition of cell proliferation in cancer cell lines (A2780, ES2, A549, H460) and normal cells (HUVEC).

Time	Sample	Cell Line				
		A2780	ES2	H460	A549	HUVEC
48 h	EHGV	313.6 μg/mL	133.1 μg/mL	105.4 μg/mL	105.4 μg/mL	113,300 μg/mL
	EHGP	177.4 μg/mL	51.4 μg/mL	360.6 μg/mL	1122 μg/mL	40,650 μg/mL
72 h	EHGV	16.92 μg/mL	137.7 μg/mL	56.51 μg/mL	22.64 μg/mL	5537 μg/mL
	EHGP	196 μg/mL	64.83 μg/mL	311.9 μg/mL	551.1 μg/mL	indeterminate

EHGV = hydroethanolic geopropolis extract of *M. fasciculata* from Viana city, Maranhão State, Brazil; EHGP = hydroethanolic geopropolis extract of *M. fasciculata* from Pinheiro city, Maranhão State, Brazil; GI_50_ values calculated by non-linear regression equation log (inhibitor) versus response—variable slope by the MTT assay. Concentration required to inhibit cell growth by 50% as determined by the dose response curve. Values are expressed as mean ± standard deviation of cytotoxicity assays (n = 4).

**Table 3:** Compounds identified in the hydroethanolic geopropolis extract produced by *M. fasciculata* stingless bee from Viana city, Maranhão State, Brazil, by *LC–ESI*/*IT–MS*/*MS.*

Compound	RT (min)	Identification	MW	[M − H]^−^ (*m*/*z*)	MS/MS Fragments (*m*/*z*)
**1**	2.8	gluconic acid	196	195	128; 177
**2**	3.1	corilagin	634	633	615; 484
**3**	15.9	taraxerone	424	423	304; 334; 406
**4**	18.2	myricetin-3-*O*-α-arabinopyranoside	450	449	430; 359; 329
**5**	19.1	prunin	434	433	313
**6**	20.5	dipterocarpol	443	442	209; 165
**7**	24	taxifolin 7-*O*-rhamnoside	450	449	405
**8**	24.7	isoschaftoside	564	563	548; 298
**9**	25.3	marsformosanone	422	421	377; 333; 297; 214; 179; 157
**10**	32	β-amyrin	427	426	232
**11**	40	typhaneoside	770	769	375; 331
**12**	44.5	3-[xyl]-28-glc-phytolaccagenin	826	825	403; 360

RT, retention time; MW, molecular weight; [M − H]^−^ molecular ion.

**Table 4:** Compounds identified in the hydroethanolic geopropolis extract produced by *M. fasciculata* stingless bee from Pinheiro city, Maranhão State, Brazil, by *LC–ESI*/*IT–MS*/*MS.*

Compound	RT (min)	Identification	MW	[M − H]^−^ (*m*/*z*)	MS/MS Fragments (*m*/*z*)
**1**	2.8	gluconic acid	196	195	128; 177
**2**	16	taraxerone	424	423	304; 334; 364; 406
**3**	17.8	dihydroquercetin-*C*-glycoside	450	449	431; 359; 329; 287; 303
**4**	18.2	dihydroquercetin-*C*-glycoside isomer	450	449	430; 359; 329
**5**	19.1	narigenin-*C*-glycoside	434	433	313
**6**	20.2	narigenin-*C*-glycoside isomer	434	433	415; 313
**7**	21	vitexin-*O*-galate	584	583	169; 313; 932; 537
**8**	22.5	pinobanksin glycosilated	436	435	270; 151; 341; 391
**9**	22,9	dihydroquercetin 3-*O*-ramnoside	450	449	303; 405
**10**	33.8	xantholaccaic acid A	521	520	262; 357; 419; 458; 502; 542
**11**	42.5	gallocatequin-xylose	438	437	305; 357; 393; 437

RT, retention time; MW, molecular weight; [M − H]^−^ molecular ion.

**Table 5:** Free binding energies obtained by molecular docking of the compounds identified in EHGV.

COX-2		NF-κB	
Ligand	ΔG_bind_ (kcal/mol)	Ligand	ΔG_bind_ (kcal/mol)
corilagin	−9.3	taraxerone	−8.4
typhaneoside	−8.8	marsformosanone	−7.7
β-amyrin	−8.7	β-amyrin	−7.4
isoschaftoside	−8.6	dipterocarpol	−6.9
3-[xyl]-28-glc-phytolaccagenin	−8.5	3-[xyl]-28-glc-phytolaccagenin	−6.9
marsformosanone	−8.5	prunin	−6.8
taraxerone	−8.3	corilagin	−6.6
prunin	−8.0	typhaneoside	−6.5
myricetin-3-*O*-α-arabinopyranoside	−7.9	isoschaftoside	−6.4
dipterocarpol	−7.7	myricetin-3-*O*-α-arabinopyranoside	−6.3
taxifolin 7-*O*-rhamnoside	−7.6	taxifolin 7-*O*-rhamnoside	−6.0
